# Can physical activity counteract the negative effects of sedentary behavior on the physical and mental health of children and adolescents? A narrative review

**DOI:** 10.3389/fpubh.2024.1412389

**Published:** 2024-08-02

**Authors:** Kun Wang, Yan Li, Hengxu Liu, Tingran Zhang, Jiong Luo

**Affiliations:** ^1^College of Physical Education, Southwest University, Research Centre for Exercise Detoxification, Chongqing, China; ^2^College of Liberal Studies (Sports Work Department), Chongqing Industry Polytechnic College, Chongqing, China

**Keywords:** children and adolescents, sedentary behavior, physical activity, physical and mental health, physiological mechanisms

## Abstract

**Background:**

The increase in sedentary behavior (SB) in children and adolescents is one of the major threats to global public health, and the relationship between physical activity (PA) and SB has always been a key topic.

**Methods:**

The literature search was conducted through PubMed, Web of Science, CNKI, Wanfang, and Scopus, and 121 pieces of literature were included in this study after screening and evaluation.

**Results:**

(1) SB caused by screen time such as mobile phones and TVs has varying degrees of negative impact on obesity, cardiovascular metabolism, skeletal muscle development, and cognitive, and psychological disorders in children and adolescents. (2) Regular physical activity could effectively prevent, offset, or improve the harm of SB to the physical and mental health of children and adolescents, mainly by reducing the incidence of obesity, and cardiovascular and metabolic risks, promoting skeletal muscle development, and improving cognitive function and mental health. (3) The mechanism of physical activity to prevent or ameliorate the harm of SB was relatively complex, mainly involving the inhibition or activation of neurobiomolecules, the improvement of blood and cell metabolic factors, and the enhancement of brain functional connectivity.

**Conclusions:**

Children and adolescents should avoid excessive SB, and through a variety of moderate to vigorous physical activity (MVPA) to replace or intermittent SB, which could effectively prevent or improve the harm of SB to physical and mental health.

## 1 Introduction

It has been reported that children and adolescents around the world have a prevailing phenomenon of excessive sedentary behavior (SB), and it was believed to reduce the physical and mental health of children and adolescents and lead to the increase of social public health burden (1, 2). The Global Action Plan on Physical Activity (2018–2030) issued by the World Health Organization (WHO) for the first time included the reduction of SB as one of the global chronic disease prevention and control strategies in 2019 (3) and has successively published global guidelines on physical activity and SB for subgroups such as children, adolescents, adults, the older adult, pregnant women, and postpartum women, as well as patients with chronic diseases or disabilities (4).

The SB usually refers to any behavior in the waking state, which is characterized by energy consumption ≤ 1.5 metabolic equivalents in sitting, reclining, or lying posture (5), and it was an inactive life state throughout the whole day, with cumulative, intermittent, and long-term characteristics. The study has shown that those who achieve moderate to vigorous physical activity (MVPA) on a daily or weekly basis were still at risk for severe SB (6), meaning that “sedentary” was not equivalent to “physical inactivity or insufficient exercise.” The data showed that more than 80% of the world's children and adolescents were physically inactive and sedentary/screen time was on the rise (4, 7), indicating that the unhealthy lifestyle of “less physical activity—how much sedentary time” was increasingly common among children and adolescents. The harm of SB to the physical and mental health of children and adolescents was well-recognized, mainly involving obesity (8), cardiovascular and metabolic diseases (9), skeletal muscle development (10), cognitive function development (11), and mental health (12). Therefore, many international researchers have explored the issues related to SB and physical activity in children and adolescents from different perspectives, but there was a lack of systematic analysis of the associated effects and mechanisms among SB, physical activity (PA), and physical and mental health. Given this, the purpose of this review is to elaborate on the harm of SB to children and adolescents from the physiological and psychological aspects, reveal the effect and mechanism of PA on improving the negative influence of SB, provide a theoretical basis and reference for promoting the formation of healthy behaviors and healthy physical and mental development of children and adolescents.

## 2 Method

### 2.1 Literature search

The literature search was conducted simultaneously in PubMed, Web of Science, CNKI, Wanfang, and Scopus citation databases. The Boolean operators were used to retrieve keywords, and they mainly include (Physical activity OR sport OR exercise) AND (Sedentary behavior OR physical activity) AND (Adolescent) AND (children). The titles, abstracts, full texts, and references of the literature were reviewed step by step, and the literature meeting the criteria was screened and supplemented, and the retrieval period was from January 1, 2000, to October 28, 2022.

Inclusion criteria: (1) The samples were children and adolescents (3–18 years old); (2) Associated with physical activity; (3) The sample meets the standard definition of SB; (4) Research indicators involve physiological or psychological aspects. Exclusion criteria: (1) The sample exceeds the specified upper and lower age limits; (2) The empirical research literature did not use physical activity or physical exercise as intervention methods; (3) Non-English or Chinese literature.

### 2.2 Search procedure

**Figure 1:**
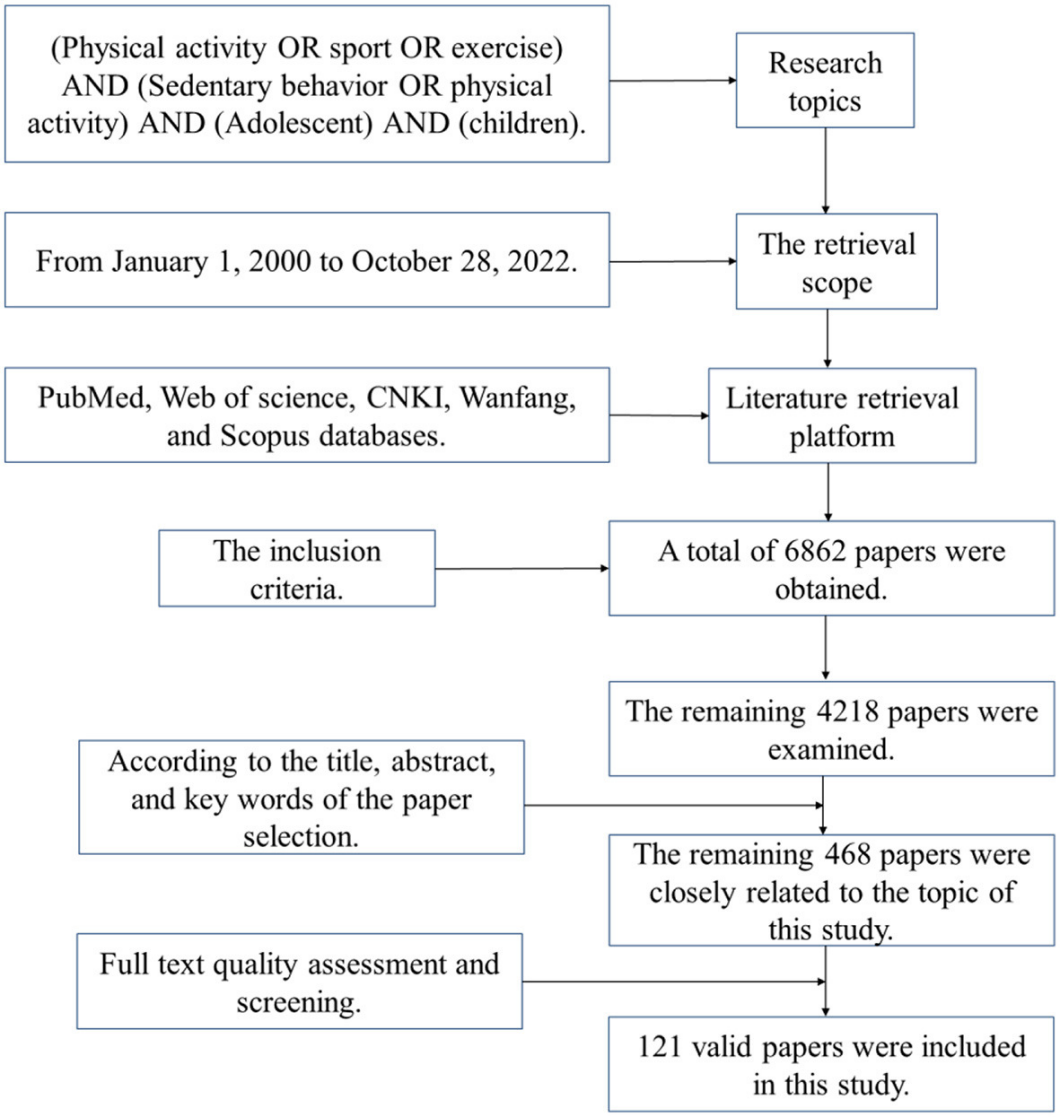
Flow chart of literature retrieval.

The literature search was divided into three steps: (1) The total of 6,862 literatures was obtained in the preliminary search, and the remaining 4,218 literatures were eliminated after the literature with similar themes and non-journal papers. (2) After carefully reading the title, abstract, and keywords of this literature, the three researchers obtained a total of 468 literatures closely related to the topic of this study after screening and statistics in Excel software; (3) Judge whether the articles fit the research theme by reading the full text, mainly excluding the polysemy of the literature. Among them, the PEDro Scale was used to examine experimental studies (such as randomized controlled trial designs) and assess their quality, and a higher score indicates better research quality. Each literature was scored independently by two researchers, if the scoring items were inconsistent, a consensus was reached after discussion. A paper with a score of 5 or more points was considered to be of high quality, while a paper with a score of 4 or less was considered to be of low quality. The literature selection was carried out by two people in parallel, and the contradictions were resolved through discussion, and the evaluation of qualified full-text articles, 121 valid literatures were finally included in this study (Figure 1).

## 3 Results

### 3.1 The relationship between SB and the health of children and adolescents

#### 3.1.1 SB and obesity

SB is closely related to the occurrence of obesity in children and adolescents, and SB dominated by screen time is an important cause of overweight and obesity, eating behavior and physical activity may be the moderating variables between SB and obesity. Studies have shown that obesity among children and adolescents was on the rise (13), and inadequate physical activity and a sedentary lifestyle were behavioral factors contributing to the rising prevalence of obesity among adolescents (14). For example, sitting for more than 4 h a day increases the risk of being overweight and obese (15). Stone et al. (16) found that the sitting time of children was positively correlated with waist circumference and body mass index (BMI), showing that with every 1 h increase in SB, waist circumference will increase by 3.4 cm and BMI will increase by 1.4 kg/m^2^. Among them, SB caused by prolonged screen time appears to be a major contributor to obesity. Such as the study has shown that most Chinese children and adolescents spend more than 2 h a day watching videos (17), and SB caused by excessive screen time will increase the obesity rate (1). A survey of 2,200 Australian adolescents aged 9–16 found a strong correlation between screen time and obesity and overweight among adolescents, especially among boys (18). Meanwhile, overweight/obese children tend to have longer screen time, and there was a positive correlation between TV watching time and overweight/obesity (19). Excessive sitting time in children and adolescents will lead to obesity and poor metabolism (8), which was manifested as those children who watch TV for more than 5 h every day were 5.3 times more likely to develop obesity than children who watch TV for <2 h, and shortening TV watching time will help promote children's physical health (20). However, some studies suggested that there was little correlation between SB and obesity in children and adolescents (21), and the obesity effect of SB may be mediated by unhealthy eating behavior and low physical activity levels (22, 23).

#### 3.1.2 SB and cardiovascular metabolism

SB is a potential cause for the development of cardiovascular and metabolic diseases at a younger age and is related to the decline of cardiovascular and metabolic levels such as inflammation, oxidative stress, and insulin secretion. In recent years, the occurrence of cardiovascular metabolic risk factors such as dyslipidemia and hypertension in children and adolescents has been on the rise (24, 25), and unhealthy lifestyle is strongly associated with chronic diseases such as hypertension and coronary heart disease (26). Studies have shown that SB is closely related to cardiovascular and metabolic diseases and their risk factors (27), the data measured by the accelerometer that people whose SB was frequently interrupted have better cardiovascular and metabolic status than those who sit for a long time (28). A study of 111 children ages 3–8 in the United States found that there was no significant correlation between children's activity and blood pressure when sedentary, but there was a significant correlation between the amount of time spent watching TV and the total screen time and the systolic and diastolic blood pressure of children (29). Meanwhile, the daily TV-watching time of adolescents was correlated with the increase in systolic blood pressure, showing a significant gender difference, and the correlation was stronger in boys (30). Further study indicated that there was a strong linear correlation between the total sedentary time and SB over 20 min and diastolic blood pressure (31), and obese children who sit and watch TV for 4 h or more per day have a 3.3 times higher risk of hypertension than children who sit and watch TV for <2 h per day (32). SB caused by watching TV for a long time or with high frequency was not only closely associated with a high-risk score of clustered cardiac metabolic diseases in children and adolescents (9, 33), but more seriously, spending more than 2 h a day on a digital screen was associated with a 5% increased risk of cardiovascular disease death in adulthood (34). The results suggested that there was a linear relationship between SB and cardiovascular disease in children and adolescents, and the time of SB more than 2 h per day was likely to increase the incidence of cardiovascular disease. The mechanism of action between sitting and cardiovascular disease can be explained as that sitting reduces the blood flow rate of the body, changes the glucose metabolism, inflammatory pathway, and oxidative stress pathway, and thus causes vascular dysfunction (35, 36). Meanwhile, SB was likely to cause pathological changes in the hemodynamic characteristics of healthy people, which were manifested as increased inflammatory response, decreased oxidation capacity of muscle mitochondria, fat oxidation, and storage capacity, resulting in decreased insulin sensitivity and insulin secretion (37), thus increasing the incidence of cardiovascular and metabolic diseases, such as diabetes and hypertension.

#### 3.1.3 SB and skeletal muscle development

The SB tends to harm healthy skeletal muscle development and is considered an adverse factor that limits normal development such as muscle strength, bone mineral content (BMC), and bone mineral density (BMD). Children and adolescents were in an important period of growth and development, during which skeletal muscle development was crucial for their healthy growth and was also a key stage for improving or promoting BMC and BMD (38). Among them, muscle strength has a significant correlation with BMC and BMD (39), and muscle endurance can have a beneficial effect on the increase of bone mass or BMD through repeated impulse loads (40). Studies have shown that SB is prone to increase the muscle tension of the soft tissues around the joints of the human body slow down the local blood circulation increase joint pressure, and lead to many serious body posture deformation problems such as pelvic forward tilt, unbalanced muscle development, and shortening of the hip flexor muscle group of the lower extremity in sedentary people (41). Meanwhile, prolonged inactivity could disrupt the balance of bone resorption and bone formation, thus negatively affecting bone health (42), while inactivity or low activity levels could also increase gene expression in skeletal muscle (43, 44). SB was also associated with decreased muscle health (maximum strength, muscle strength, and local muscle endurance) (45). Grontved et al. (46) found that screen time was negatively correlated with an individual's isometric trunk muscle strength, so limiting screen time might be beneficial to improving or maintaining the isometric trunk muscle strength. Researchers have found that time previously spent on physical activity may be replaced by the time children spend on SB (such as watching television or playing computer games) which may adversely affect children's bone health (10) and that longer sedentary time was significantly associated with lower muscle strength in children (47). A cross-sectional study showed that BMC of the proximal femoral was inversely associated with adolescents' self-reported total screen time, but not computer time (48), and that total BMC of the body was significantly reduced when more than 3 h of TV viewing per day was observed (49). However, other studies have taken a different view, such as that there was no relationship between the total BMC of adolescent girls and TV watching time (50). There was no strong association between self-reported or objectively measured screen time and bone parameters such as skeleton structure, BMD, or strength in healthy adolescents, and the researchers suggested that differences in measurement location may be responsible for the differences in the effects of sedentary time on bone results (51).

#### 3.1.4 SB and cognitive development

The cognitive function of children and adolescents was the most plastic (52), however, under the influence of certain unhealthy behaviors (such as SB), it may adversely affect the development of cognitive function (11, 53). Studies have shown that excessive sitting time in adolescents was not conducive to the development of executive functions such as emotional control and cognitive conversion (54), and SB formed by watching TV for a long time was negatively correlated with cognitive function (11). Compared with children who watch less TV, those who watch more TV have lower executive function (55). SB could negatively affect overall cognitive function throughout the life cycle, but it was not known exactly which components of cognitive function were involved (56). Coelho et al. (57) pointed out that individuals with less SB perform better on executive function and memory tasks than their sedentary peers. Meanwhile, the relationship between SB and cognitive function may vary according to different activities (such as screen time, reading, learning, and driving) that individuals engage in during sedentary conditions (57), suggesting that the relationship between SB and cognitive function is not simple or linear. Such as children and adolescents daily learning and attending classes belong to learning sedentary behavior (58), which was believed to promote the development of children's executive function (59), but there is currently no corresponding causal association mechanism. Among adults, SB based on computer or internet use was positively associated with improved cognitive function (11, 60). The result suggested that not all SB were associated with reduced cognitive function and were influenced by the individual's environment or social background (55), such as the effects on cognition were different when an individual was engaged in a passive sedentary activity (such as watching television) or a sedentary activity that stimulates cognition (such as using a computer or studying). On the one hand, the passive SB caused by screen time such as watching TV was more likely to inhibit the development of cognitive function in children and adolescents, which may be interpreted as the long-term SB damages the body's glucose and lipid metabolism (42), which was considered to be a risk factor for cognitive decline and all-cause dementia (61, 62). Meanwhile, with the increase in sitting time, the body will correspondingly show an increase in white matter volume, a decrease in brain-derived neurotrophic factor (BDNF) level, and abnormal cerebral blood flow, which will lead to a decline in cognitive function (53). On the other hand, the learning SB formed by learning, reading, or watching the computer could promote the cognitive ability of children and adolescents to some extent, which may be related to the rational allocation of cognitive resources such as attention regulation, cognitive conversion, and emotional control.

#### 3.1.5 SB and mental health

As was known to all, children and adolescents are in an important period of sound personality and rapid psychological development, and once psychological problems occur, they will bring great mental pain to themselves and damage corresponding social functions (63). Studies have found that SB in children and adolescents was associated with poor internalization problems and prosocial behavior (64), and SB itself may harm the emotional state, thus impeding positive motivation (65). Meanwhile, excessive sitting time will affect the mental health of children and adolescents, leading to an increase in the incidence of depression, mood disorders, and other mental diseases, which is not conducive to the development of their emotional control function (54, 66), and long-term watching TV, using the computer, and playing video games was not conducive to the development of prosocial behaviors of children and adolescents (33, 67). The negative effects of SB on prosocial behavior increased with the increase in screen time when the focus was on TV and video games (68), and higher screen time (video games/TV watching) was significantly associated with lower self-esteem and bullying behavior among adolescents (69). A systematic review has also reported an association between screen time and mental health indicators in children and adolescents, including hyperactivity/inattention problems, internalization problems, and perceived quality of life (70), and SB caused by long-term screen time was positively correlated with depression and anxiety disorders (12, 71). Interestingly, a study has found that the time of watching TV was associated with higher depressive symptoms, while internet use and reading were associated with lower depressive symptoms (55), but corresponding research evidence was lacking in children and adolescents. In summary, SB is related to the mental health of children and adolescents, and whether this correlation is negative or positive is easily affected by factors such as the type of and the time of SB. For example, passive SB such as watching TV or playing games harms the mental health of children and adolescents, and appropriate use of the internet may be beneficial to promoting social interpersonal, relieving inner depressive mood, and enhancing the level of mental health (see Table 1 for some details).

**Table 1:** Effects of SB on the physical and mental health of children and adolescents.

**References**	**Participants' age (years)**	**Sedentary types**	**Study design**	**Measurement indicators**	**Conclusions**
Stone et al. (16)	11	—	Physical activity and SB of 856 children (Data from Project BEAT) were monitored with accelerometers, and outdoor play was assessed through parental reports and classified as low, moderate, or high.	Sedentary time, physical activity, outdoor games, and BMI.	Sedentary time was positively correlated with waist circumference and BMI, showing that every 1 h increase of SB increases waist circumference by 3.4 cm and BMI by 1.4 kg/m^2^.
Tremblay et al. (1)	5–17	Sedentary, Watching TV, playing video games, or non-school computer use	Systematic review and meta-analysis involving 232 studies.	Body composition, physical health, metabolic syndrome, cardiovascular disease, self-esteem, behavioral/prosocial behavior, and academic achievement.	Reducing any type of sedentary time has been linked to lower health risks, with watching more than 2 h of TV a day associated with lower physical and mental health, and reducing sedentary time associated with lower BMI.
Al-Ghamdi et al. (19)	9–14	Watching TV	Using a case-control study design, 200 obese school-age children were included in the experimental group and 197 non-obese children were included in the control group. Participants' sedentary and screen time was assessed through questionnaires.	The time of watching TV, BMI.	Watching TV was an important risk factor for obesity in school-age children, and increased time TV watching was positively correlated with the risk of childhood obesity.
Carson et al. (9)	5–17	Watching TV, using the computer, or playing video games	Systematic review and meta-analysis involving 235 studies.	Body composition, metabolic syndrome/cardiovascular disease risk factors, behavioral/prosocial behavior, academic achievement, physical fitness, and self-esteem.	Excessive duration and frequency of screen time were associated with varying degrees of negative effects on body composition, cardiometabolic risk, prosocial behavior, physical fitness, and self-esteem, while longer hours spent reading and doing homework were associated with higher academic achievement.
Martinez-Gomez et al. (29)	3–18	Watching TV, using the computer, or TV + computer's screen time	The relationship between variables was established when 111 participants were assessed for obesity using dual-energy X-ray absorptance and were given objective measures of sedentary by wearing accelerometers for 7 consecutive days and screen time was obtained from parental reports.	Obesity, body composition, SB, time of watching TV, computer time, and screen time (TV + computer).	SB, specifically watching TV and screen time, was associated with children's blood pressure, not body composition.
Pardee et al. (32)	4–17	Watching TV	The relationship between variables was examined by measuring BMI and blood pressure in 546 obese children, and by subjectively reporting sedentary TV viewing time by parents or children.	BMI, blood pressure, and time of watching TV.	The time of watching TV was associated with high blood pressure and the severity of obesity, and sitting and watching TV for more than 2 h a day greatly increases the risk of high blood pressure in children.
Heidemann et al. (10)	7–12	—	Physical activity and SB in 742 children were monitored for 7 days using a triaxial accelerometer, and BMC, BMD, and BA were measured using whole-body DXA scans.	Sedentary time, physical activity, BMC, BMD, and BA.	SB could adversely affect bone health such as BMC, BMD, and BA in children.
Cieśla et al. (47)	6–7	Play video games	The parents of 25,816 children were surveyed by questionnaires and the physical fitness indicators were measured.	BMI, trunk strength, lower limb strength, and upper body strength.	BMI and SB were factors that limit normal levels of healthy physical fitness components, and longer sedentary time was associated with lower muscle strength.
Huber et al. (59)	2–3	Watching TV	96 children were asked to watch educational apps, educational TV, or cartoon TV on iPad, and their executive function was measured before and after the experiment.	Executive functions such as working memory, response inhibition, and task switching.	Long-term TV viewing was associated with lower levels of executive functioning, but interactive educational content had a positive effect on children's working memory while watching TV, and the media experience was more important than the screen itself.
Fairclough et al. (64)	9–13	—	The triaxial accelerometer was used to monitor the daily behavior and activity level of 359 children and adolescents for 7 consecutive days, and their physical and mental health indicators were measured, to establish the relationship between variables.	BMI, sleep duration, self-esteem, mood, prosocial behavior, and cognitive function.	The time of SB was associated with greater internalization problems and worse prosocial behavior.
Wen et al. (65)	8–12	Watching TV, watching videos, or playing video games	The ecological transient assessment survey was used to guide the emotional state and life behavior of the participants. Based on the subjective report of the stress and emotional state of the participants, the accelerometer was used to measure their physical activity and SB.	Stress, mood, sedentary time, and physical activity.	SB could negatively affect the mental health of children and adolescents, thereby hindering the development of positive motivation.

DXA, dual-energy X-ray absorption assay; BMD, bone mineral density; BMC, bone mineral content; and BA, bone area.

### 3.2 PA could regulate the effects of SB on the physical and mental health

**Figure 2:**
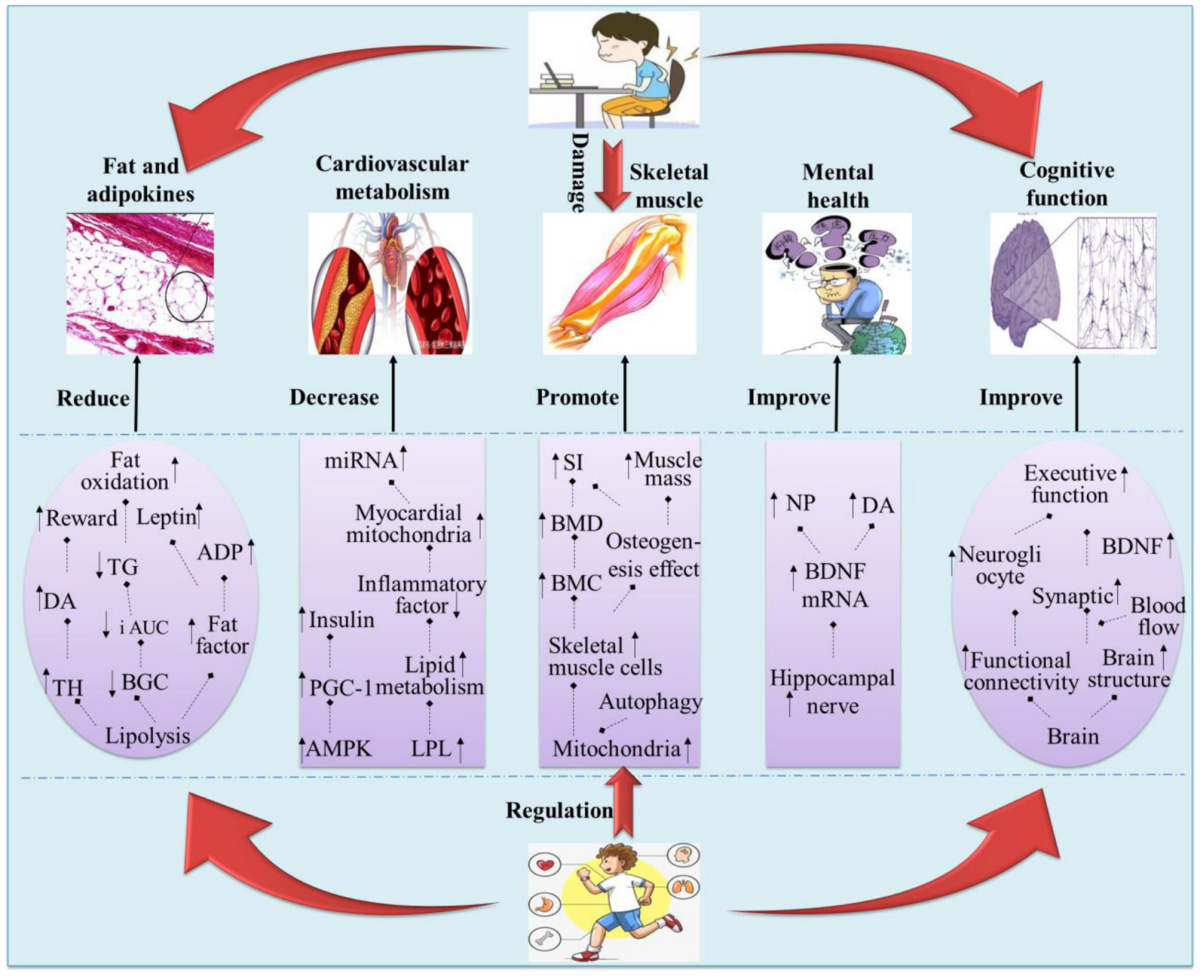
Possible pathways by which physical activity mediates the SB in children and adolescents. “
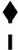
” indicates a potential path relationship between two words; “↑” indicates increase or promote; “↓” indicates inhibit or decrease.

PA was considered to have regulatory benefits on the physical and mental health of children and adolescents to varying degrees, and severe SB not only reflects the interruption of the continuity of PA but also predicts higher health risks (28). The relationship between SB and physical activity has become a focus of public health researchers, and high levels of physical activity and low levels of SB were closely related to optimal physical and mental health (72). Among them, the iso-temporal substitution model (ISM) was generally considered to be able to better interpret the relationship between the two (73–75). However, the replacement of SB by PA was mostly at the phenomenon level, and few studies focused on how PA regulates the influence of SB on the physical and mental health of children and adolescents, and there was a lack of systematic analysis of the relationship among PA, SB, and physical and mental health from the mechanism level (see Figure 2 for relevant details).

#### 3.2.1 The regulating effect of PA on the physiological function of SB

##### 3.2.1.1 Reduce the incidence of obesity

Reviewing the above, SB dominated by screen time was likely to lead to overweight or obesity in children and adolescents, while appropriate physical activity could reduce the increase in childhood obesity rate (76), improve body composition, and reduce body fat content, to prevent the occurrence of overweight and obesity (77). So can the negative effects of SB on body fat be interrupted or offset by being physically active? Studies (78, 79) have shown that both low-intensity physical activity (LPA) and MVPA can effectively regulate the association between sedentary and obesity indicators in children and adolescents, and reduce the incidence of overweight or obesity. However, compared with LPA, MVPA may be more beneficial in reducing the incidence of obesity. The study has found that obesity in children and adolescents could be effectively controlled or alleviated when ISM was used to replace LPA and SB with MVPA equivalent time (10–60 min) (73). Meanwhile, replacing LPA, SB, and sleep with MVPA, as well as replacing sedentary behavior with LPA could reduce the risk of obesity, while replacing MVPA, LPA, and sleep with SB could increase the risk of obesity (74). So why does substituting PA for SB reduce the incidence of obesity? This may be related to the fact that physical activity reduces blood glucose concentration (BGC) and fat content, as well as increases fat oxidation. For example, Kim et al. (80) showed that during a 9 h sedentary period, intermittent LPA walking every 60 min (9 times, accumulated for 3.5 h) would significantly reduce the BGC of the next day after meals and decreased area increase under the curve (i AUC) in Triglyceride (TG) and increased fat oxidation. A similar effect was found in moderate PA, such as 30–60 min/time and 3–5 times per week, which was believed to reduce the total body fat and visceral fat content of children and adolescents (81) and promote the decomposition and reduction of fat mass, significantly increase the level of serum leptin and Adiponectin (ADP) (82), thus reducing the incidence of obesity after sitting for a long time. Moreover, the decrease in BGC and TG and the increase in fat oxidation were also thought to be related to it (80). PA also promotes the synthesis of Dopamine (DA) by upregulation of tyrosine hydroxylase (TH) in the midbrain-striatum system, regulates reward function by reducing the response of midbrain DA neurons to exogenous stimuli, improves adverse behavior, and increases PA levels to achieve the role of weight control (83).

##### 3.2.1.2 Reduce cardiovascular and metabolic risk

A study from the United States found that there was no significant correlation between interrupted SB and cardiovascular risk factors (84), indicating that children and adolescents' cardiovascular and metabolic risks cannot be reduced simply through intermittent sedentary, and appropriate PA may be a more important way (85). Studies have shown that, given the non-linear dose-response relationship between sitting and cardiovascular diseases, it was necessary to avoid sitting while taking more PA per week than recommended by WHO, to reduce the health hazards of sitting to some extent (86), especially in children and adolescents (87, 88). LPA was a favorable factor for cardiometabolic biomarkers (33), and intermittent LPA could regulate the harm of prolonged sitting and thus enhance the cardiometabolic health of individuals (89). The cohort studies have found that the replacement of SB by LPA or MVPA reduces type 2 diabetes risk, cardiovascular mortality, and all-cause mortality (90). In the United States, MVPA was seen as an attractive non-pharmacological intervention strategy for the prevention and management of cardiovascular disease (91). Among people who were sedentary for more than 5 h per day, reassigning time from sedentary to PA of any intensity could reduce the incidence of cardiovascular disease, suggesting that different levels of physical activity were significantly associated with reduced risk of cardiovascular disease (92). Ekelund et al. (93) suggested that physical activity could alter the association between sitting and negative health outcomes and that higher levels of PA could reduce or eliminate the risk of cardiovascular disease induced by SB. The reason was that SB could reduce the activity of lipoprotein lipase (LPL) in slow muscle fibers, while exercise or PA could increase the activity of LPL in fast muscle fibers, and the lipoprotein metabolism was directly regulated by LPL, plays a central role in regional lipid deposition and plasma lipoprotein distribution, and was associated with the clinical prognosis of cardiovascular diseases and chronic metabolic diseases (85). PA could promote PGC-1 gene expression through AMPK, thus improving insulin resistance (94, 95), moreover, exercise could also reduce the inflammatory response, improve vascular endothelial and myocardial mitochondrial function, along with miRNA activation, improve energy utilization and cardiovascular metabolism (96).

##### 3.2.1.3 Promote skeletal muscle development

Studies have suggested that PA may counteract the adverse effects of SB on bone health by improving the incidence of low BMC in children and adolescents associated with prolonged watching TV (49), and was significantly correlated with the increase in BMD (97). The increase in bone trabecula and bone strength in the distal femur was significantly related to PA, which maximizes muscle mass and bone strength and may counteract the development of osteoporosis and bone vulnerability in adulthood (98). This was because appropriate exercise or PA could improve the autophagy level of mitochondria, inhibit apoptosis or accidental death of skeletal muscle and cardiomyocyte (99, 100), and regulate fat, skeletal muscle, and soft tissue, thus exerting direct and indirect influences on skeletal muscle development (101). At present, the promoting effect of MVPA on skeletal muscle development seems to be recognized and supported by more scholars (102). An accelerometer-based study showed that MVPA reduced the adverse effects of children's SB and sedentary time on bone stiffness index (SI), and MVPA participation was more effective than LPA in increasing children's SI (103). Harvey et al. (104) found that 10 min of additional MVPA per day promoted a 1.4% increase in BMC in children, and increasing childhood PA levels and calcium intake may help optimize bone mass gain. Meanwhile, the osteogenic effect of VPA decreased with the decrease in activity intensity. Increasing VPA for 10 min a day could increase SI by nearly 2%, while the same MPA only increases SI by 1% (103). Studies have found that participation in VPA was more significantly correlated with bone strength indicators in children than LPA, MPA, or MVPA (97, 105), and VPA was believed to optimize bone development early in life, thereby preventing age-related bone loss and osteoporotic fractures (106). The result suggested that there was a dose effect between physical activity and skeletal muscle development, and the greater the activity, the better the improvement effect. Moreover, shorter negative heavy PA could enhance the bone mass, density, and structure of different bone parts in children and adolescents (107), which could offset the potential harm of sedentary time to bone health through a small amount of negative heavy PA (51).

#### 3.2.2 Moderating effects of PA on mental cognition of SB

##### 3.2.2.1 Improve cognitive function

Studies have shown that when individuals have a low level of PA, past SB has a negative predictive effect on cognitive inhibition, while PA may have a positive protective effect on cognitive impairment caused by SB (108). Compared with sedentary children, children with high levels of PA have better executive function development, which was attributed to the fact that physically active children were more able to control their activities and behaviors (109). Fairclough et al. (64) believed that reallocating more sedentary or LPA time to MVPA may be more beneficial to the improvement of executive function in children and adolescents. Similarly, reducing discretionary sedentary time to <2 h per day, coupled with ≥150 min of MVPA per week, maybe the best way to promote healthy cognition (56). This might be explained by PA through the optimization of the brain cell and molecular level, brain structure, and function of physiological mechanism, improved cell blood flow environment, promotion the glial cells regeneration, improved synaptic plasticity, and increased neurotransmitter levels, improved children's working memory, perception, movement coordination, and executive function (110). Ishihara et al. (111) also reported that a single 50-min tennis game class could significantly improve the executive function (total scores of inhibitory control, working memory, and cognitive flexibility) of children aged 6–12, and the effect was better than repetitive exercise. The results suggested that regular PA may at least mediate, if not eliminate, the potential negative relationship between SB and cognitive function in children and adolescents, and the moderating effects may depend to some extent on the intensity, time, and frequency of PA.

##### 3.2.2.2 Improve mental health

A study of 2,464 students aged 12–15 in Norway indicated that reducing SB such as watching TV and engaging in more VPA was key to relieving stress in students' lives and preventing depression (112). Excessive screen time and insufficient PA will increase the incidence of various mental diseases in children and adolescents, and reducing screen time and increasing PA participation time were important ways to prevent depressive symptoms and related mental diseases (113, 114). When MVPA was used to replace LPA and SB for 10–60 min, the mental illness of children and adolescents could be effectively controlled or alleviated (115). Similarly, the use of MVPA, such as active recess and walking/running, to replace SB or LPA has also been found to benefit children's self-esteem, mood, and prosocial behavior to a greater extent (64). Meanwhile, PA intervention in the school environment has a significant positive impact on the improvement of children and adolescents' resilience, anxiety (116), and subjective wellbeing (117), which may be due to the combined effect of PA and sedentary discontinuity. The regulatory benefits of PA in mood may be related to the hippocampus and neurotransmitters because the hippocampus is the central brain region that regulates anxiety (118), and PA could effectively promote the growth of the hippocampus nerve (119). Animal experiments have shown that hippocampal BDNF mRNA level increases and anxious behavior in behavioral experiments was significantly reduced when mice ran wheel exercise (120), and regular PA could also increase the release of endorphin (NP), thereby reducing depression, anxiety, and other negative emotions (121). Therefore, PA may eventually moderate the negative effects of sitting on the mental health of children and adolescents by improving psychological indicators such as mental illness and negative emotions.

## 4 Discussions

In this literature review, we searched and identified 123 published academic papers that explored the relationship between SB, PA, and physical and mental health in children and adolescents based on the logic of the “phenomenal association mechanism.” Although some of the current research was controversial, overall, SB in children and adolescents was harmful to their physical and mental health, and the idea that PA could effectively regulate this harm was supported by the majority of research (51, 56, 103).

### 4.1 The harm of SB

It was well-known that sedentary behavior (energy expenditure ≤ 1.5 MEE) was detrimental to the physical health of individuals, especially for children and adolescents who were in the formative stages of growth and mental health. Through literature review, it was found that screen time was an important factor leading to SB in children and adolescents, and long-term passive SB (such as watching TV, and playing video games) was a risk factor for their obesity rate, the incidence of cardiovascular and metabolic diseases, and not only affect the development of skeletal muscle but also hinder the development of cognitive function and mental health. It was important to note that not all SB have negative effects on physical and mental health, and may vary depending on the type of activity an individual was engaged in during a sedentary period. For example, within a reasonable time range, learning-related SB could be beneficial to the development of cognitive function and mental health of children and adolescents to a certain extent (58, 59), including the rational allocation of cognitive resources such as attention regulation, cognitive transformation, and emotional control, but the corresponding mechanism still needs to be explored. Meanwhile, scientific questions such as whether there is a synergistic effect of SB on different health indicators, the dose-effect of sedentary time, and how to regulate the effects of SB on physical and mental health still need to be further explored.

### 4.2 The regulation of PA

PA has long been recognized as a green, environmentally friendly, and effective way to promote health, with unique value in preventing or improving chronic diseases. On this basis, this study found that the use of PA (especially MVPA) to replace SB was an important way to prevent or improve the physical and mental health diseases caused by excessive sedentary of children and adolescents. To a certain extent, physical activity can effectively interrupt, offset, and improve the harm of SB on the body shape, cardiovascular metabolism, and skeletal muscle development of children and adolescents, and play an important regulatory role in the relationship between SB and health. For example, PA after SB not only promotes the breakdown of fat mass, alleviates the imbalance of adipokines, and increases fat oxidation, but also improves the function of vascular endothelium and myocardial mitochondria, reduces inflammation, and accompanies miRNA activation, improves energy utilization and cardiovascular metabolism. Equally important, PA may ameliorate the risk of reduced BMC and increased BMD in children and adolescents due to SB, as well as promote healthy cognitive and psychological development by regulating brain neurotransmitters and functional connections. However, there was still a lack of direct evidence on whether the health promotion benefits of PA were caused by the increase in PA or the decrease in SB. Meanwhile, more empirical studies are needed to support the dose-effect and regulatory mechanism of PA on SB.

### 4.3 Limitation

This study systematically integrated and sorted out the studies related to SB and PA in children and adolescents, but the existing studies still cannot effectively solve many relevant hot issues. For example: (1) How to accurately define and distinguish the learning or passive SB of children and adolescents, as well as the best time for intermittent SB, and the future can further explore the positive or negative impact of different types of SB on individual physical and mental health, and reveal the causal mechanism. (2) This study did not consider the individual differences in the age and health status of children and adolescents in terms of SB and physical activity. Therefore, follow-up studies can deeply explore the physiological and psychological effects of SB on children and adolescents with different population differences, as well as the mechanism of action and dose-effect differences of physical activity amount to reduce the harm of SB. (3) Further research may also explore how to measure the fitness of SB and physical activity guidelines for children and adolescents with different demographic characteristics (such as age, sex, and physical fitness status). These key issues need to be enriched and improved by subsequent research. Moreover, due to the regional differences between children and adolescents in different countries, how to develop more scientific, special, and targeted physical activity guidelines needs to be further explored.

## 5 Conclusions

The long-term passive SB caused by screen time was likely to increase the rate of obesity and cardiovascular and metabolic diseases in children and adolescents and limit the development of skeletal muscle, cognitive function, and mental health shaping and development. Interestingly, regular PA could, to some extent, effectively prevent, offset, or ameliorate the harmful effects of SB on the physical and mental health of children and adolescents. It has the physiological benefits of reducing the incidence of obesity and cardiovascular and metabolic risk and promoting the development of skeletal muscle, as well as the psychological benefits of improving cognitive function and mental health. However, there was still little direct evidence on whether the health-promoting benefits were caused by increased PA or a decline in SB. It was worth noting that the related mechanisms of PA to improve the harm of SB were relatively complex, mainly involving the inhibition or activation of neuro biomolecules, improvement of blood and cell metabolic factors, and enhancement of brain functional connectivity. Children and adolescents should avoid excessive SB and substitute or interrupt SB with different forms of PA, which could effectively prevent or improve the harm of SB to physical and mental health. Among them, the recommended PA consists of two parts: daily PA (60 min or more, moderate to high intensity, daily frequency) and additional PA (high intensity and strong skeletal muscle activity 3 or more times per week).

## Author contributions

KW: Conceptualization, Methodology, Writing – original draft. YL: Conceptualization, Resources, Writing – original draft. HL: Data curation, Validation, Writing – review & editing. TZ: Supervision, Writing – review & editing. JL: Project administration, Writing – review & editing.
